# PACSIN1 promotes immunosuppression in gastric cancer by degrading MHC-I: The roles of PACSIN1 in gastric cancer

**DOI:** 10.3724/abbs.2024059

**Published:** 2024-05-31

**Authors:** Zhu Liu, Xin Li, Ali Muhammad, Qiannan Sun, Qi Zhang, Yang Wang, Yong Wang, Jun Ren, Daorong Wang

**Affiliations:** 1 The Yangzhou School of Clinical Medicine of Nanjing Medical University Yangzhou 225001 China; 2 Clinical Medical College Yangzhou University Yangzhou 225001 China; 3 Northern Jiangsu People’s Hospital Yangzhou 225001 China; 4 General Surgery Institute of Yangzhou Yangzhou University Yangzhou 225001 China; 5 Yangzhou Key Laboratory of Basic and Clinical Transformation of Digestive and Metabolic Diseases Yangzhou 225001 China; 6 Department of Pharmacy Clinical Medical College Yangzhou University Northern Jiangsu People’s Hospital Yangzhou 225001 China

**Keywords:** gastric cancer, PACSIN1, autophagy, MHC-I, CD8 ^+^ T cell

## Abstract

Gastric cancer (GC) is a common gastrointestinal system malignancy. *PACSIN1* functions as an oncogene in various cancers. This study aims to investigate the potential of PACSIN1 as a target in GC treatment. Gene expression is determined by RT-qPCR, immunofluorescence staining, and immunohistochemistry assay. FISH is performed to determine the colocalization of PACSIN1 and the major histocompatibility complex (MHC-I). Cytokine release and cell functions are analyzed by flow cytometry. *In vivo* assays are also conducted. Histological analysis is performed using H&E staining. The results show that PACSIN1 is overexpressed in GC patients, especially in those with immunologically-cold tumors. A high level of PACSIN1 is associated with poor prognosis. PACSIN1 deficiency inhibits autophagy but increases antigen presentation in GC cells. Moreover, PACSIN1 deficiency inhibits the lysosomal fusion and selective autophagy of MHC-I, increases CD8 ^+^ T-cell infiltration, and suppresses tumor growth and liver metastasis *in vivo*. Additionally, *PACSIN1* knockout enhances the chemosensitivity of cells to immune checkpoint blockade. In summary, PACSIN1 mediates lysosomal fusion and selective autophagy of MHC-I and suppresses antigen presentation and CD8 ^+^ T-cell infiltration, thus inhibiting antitumor immunity in GC.

## Introduction

Gastric cancer (GC) ranks the fifth most common malignant cancer [ 1, 2] . Compared to that in 2014, the mortality of GC is increased by 1.4% [3]. In China, the incidence rate of GC ranks second among all cancers [4]. Although great advances have been made in GC therapies in recent decades, the clinical outcomes are still unsatisfactory [5]. The 5-year mortality rate of GC is still over 30% [6]. This is because most patients are diagnosed at an advanced stage with acquired chemoresistance [ 7– 10] . Therefore, identifying the molecular mechanisms underlying the pathogenesis of GC may provide novel strategies for the treatment of GC.

Immune checkpoint blockade (ICB) strategies, especially anti-PD-1 and anti-PD-L1 antibodies, have demonstrated remarkable efficacy in suppressing malignancy by stimulating antitumor T-cell immunity [ 8, 11, 12] . PD-1 and its ligand inhibit antitumor T-cell immunity in the following two ways: 1) PD-L1 downregulates MHC-I, which decreases antigen presentation in tumor cells and dampens CD8 ^+^ T-cell killing ability; 2) PD-1-expressing CD8 ^+^ T cells are contributes to T cell exhaustion [ 13– 15] . Therefore, a PD-1- or PD-L1-mediated decrease in terminal effector T cells induces immune silencing. However, anti-PD-1 and anti-PD-L1 antibodies increase the level of MHC-I on the surface of tumor cells, which suppresses immune evasion in GC [16]. Therefore, MHC-I may be a potential target for increasing the sensitivity of ICB strategies.

PACSIN1, a member of the PACSIN protein family, is the key regulator of the synaptic vesicle transport cycle and receptor-mediated endocytosis [17]. PACSIN1 contains an N-terminal F-BAR domain and a C-terminal SH3 domain [18]. F-BAR domains promote the assembly of microtubules, while the SH3 domain modulates membrane shaping and reconstruction [19]. Moreover, PACSIN1 is neurospecific. Abnormally expressed PACSIN1 is associated with the pathogenesis of long-term depression in the cerebellum and Huntington’s disease [ 20, 21] . Moreover, PACSIN1 is overexpressed in multiple types of cancers. For instance, overexpressed PACSIN1 is associated with poor overall survival in luminal breast cancer patients [22]. Overexpressed PACSIN1 predicts the metastasis of GC [23]. However, the underlying molecular mechanisms are still unclear.

This study aimed to investigate the role of PACSIN1 in the tumor microenvironment in GC. Targeting PACSIN1 may enhance the chemosensitivity of ICB strategies.

## Materials and Methods

### Specimen

Clinical samples were collected from GC patients ( *n*=62) hospitalized in North Jiangsu People’s Hospital from January 1, 2022 to December 31, 2022. The GC tissues and adjacent normal tissues were immediately stored in liquid nitrogen at –80°C. All participants who previously received chemotherapy or radiotherapy were excluded. This study was approved by the Ethical Committee of North Jiangsu People’s Hospital and consent forms were signed by all patients.

### Immunohistochemistry (IHC) assay

The slides were fixed in paraffin, deparaffinized and immersed in EDTA buffer (P0085; Beyotime, Shanghai, China). After being blocked with 1% BSA (36100ES25; YESEN, Shanghai, China), the sections were incubated with primary antibodies against PACSIN1 (HPA028852; 1:50; Sigma-Aldrich, St Louis, USA). Then, the sections were incubated with the corresponding HRP-conjugated secondary antibodies (ab6721; 1:1000; Abcam, Cambridge, USA). Afterwards, the sections were counterstained with DAPI staining solution (C1005; Beyotime) and DAB solution (P0203; Beyotime) according to the manufacturer’s protocol. Subsequently, the sections were imaged using a microscope (Nikon, Tokyo, Japan) at 400× magnification.

### Cell culture

The human GC cell lines MKN-45 and AGS, the mouse GC cell line MFC, and the human normal gastric epithelial cell line GES-1 were obtained from ATCC (Manassas, USA). All these cells were cultured in RPMI-1640 medium (SH30096.01; HyClone, Carlsbad, USA) supplemented with 10% FBS (SH30070.03IH25-40; HyClone) at 37°C in an incubator (Thermo Fisher Scientific, Waltham, USA) with 5% CO _2_.

### Cell transfection

*B2M* shRNA (5′-GCCTGAGTGTATCTCAGATCC-3′) and its negative control (NC: 5′-TGCCCGACAACCACTACCTGA-3′) were provided by GenePharma (Shanghai, China). Mouse GC cells (MFCs) were transfected using Lipofectamine ^®^ 2000 reagents (11668019; Thermo Fisher Scientific) and cultured for 6 h at 37°C in 5% CO _2_. Afterwards, cells were incubated for 48 h after transfection.

### Western blot analysis

Proteins were isolated from all cell samples, GC tissues and adjacent normal tissues using RIPA buffer (9800; Cell Signaling Technology, Beverly, USA), separated by 8% SDS-PAGE (P0688; Beyotime), and transferred to a PVDF membrane (ISEQ07850; Millipore, Billerica, USA). The membranes were blocked with TBST buffer (60145ES76; YESEN) containing 5% skimmed milk (36120ES60; YESEN). All membranes were then incubated for 1.5 h at room temperature with the indicated primary antibodies against a range of proteins, including anti-PACSIN1 (HPA028852; 1:3000; Sigma-Aldrich), anti-MHC-I (SAB5703013; 1:2000; Sigma-Aldrich), anti-B2M (SAB2105109; 1:2000; Sigma-Aldrich), anti-CD47 (SAB5700964; 1:2000; Sigma-Aldrich), anti-ANXA1 (SAB5700044; 1:2000; Sigma-Aldrich), anti-Junb (SAB5701565; 1:2000; Sigma-Aldrich), anti-Fos (SAB2100833; 1:2000; Sigma-Aldrich), anti-Jund (SAB4501615; 1:2000; Sigma-Aldrich), anti-Fosb (AV32519; 1:2000; Sigma-Aldrich), and anti-β-actin (A5316; 1:2000; Sigma-Aldrich). The membranes were then incubated with the corresponding goat-anti-rabbit secondary antibodies (ab6721; 1:20,000; Abcam) or goat-anti-mouse (ab205719; 1:20,000; Abcam) for 1 h at room temperature. The blots were then detected and analyzed using the FluorChem System (ProteinSimple v.3.5.0; Bio-Techne, San Francisco, USA).

### *PACSIN1* ^‒/‒^ cell generation

MFC cells (2×10 ^5^) were inoculated in a 6-well plate and cultured overnight at 37°C in an incubator. Mouse PACSIN1 nickase plasmids targeting *PACSIN1* loci in mouse GC cells were purchased from GenePharm (Shanghai, China). Then the mouse PACSIN1 nickase plasmids were transfected into MFC cells using Lipofectamine ^®^ 2000 reagents according to the product’s instructions. After transfection, the cells were selected in medium supplemented with 2 μg/mL puromycin (SC-108071C; Santa Cruz Biotechnology, Santa Cruz, USA).

### RT-qPCR

Total RNAs were isolated from MKN-45, AGS and MFC cells, human gastric tissues and mouse gastric tissues using TRIeasy Total RNA Extraction Reagent (Tcm Free, 19202ES60; YESEN). cDNA synthesis was carried out using a RevertAid First Strand cDNA Synthesis kit (K1622; Thermo Fisher Scientific). To determine mRNA expression, PCR was performed using the FastStart Universal SYBR Green Master (Rox) kit (04913914001; Roche, Basel, Switzerland). The cycling conditions were as follows: 10 min at 95°C, followed by 40 cycles of 95°C for 10 s, 60°C for 20 s and 72°C for 42 s. Relative mRNA expression was calculated using the 2 ^‒ΔΔCq^ method. mRNA expression was normalized to that of *GAPDH*. The sequences of the primers used are shown in Table 1.

**Table:** **Table 1** The sequences of the primers used in PCR

Name	Sequence (5′→3′)
Homo *PACSIN1*	F: CCACCGTCTATGCAACGACC
	R: CAGGCTGCCATACTGTGGG
Mus *MHC-I*	F: GAGACACAGGTCGCCAAGAAC
	R: CGCTGGTAAGTGTGAGAGCC
Mus *B2M*	F: TTCTGGTGCTTGTCTCACTGA
	R: CAGTATGTTCGGCTTCCCATTC
Mus *CD74*	F: AGTGCGACGAGAACGGTAAC
	R: CGTTGGGGAACACACACCA
Mus *ANXA1*	F: AAGCAGGCCCGTTTTCTTGAA
	R: GCAACATCCGAGGATACATTGA
Mus *Junb*	F: CTATCGGGGTCTCAAGGGTC
	R: CTGTTGGGGACGATCAAGC
Mus *Fos*	F: CGGGTTTCAACGCCGACTA
	R: TGGCACTAGAGACGGACAGAT
Mus *Jund*	F: GAAACGCCCTTCTATGGCGA
	R: CAGCGCGTCTTTCTTCAGC
Mus *Fosb*	F: CCTCCGCCGAGTCTCAGTA
	R: CCTGGCATGTCATAAGGGTCA
Homo *GAPDH*	F: TGTGGGCATCAATGGATTTGG
	R: ACACCATGTATTCCGGGTCAAT
Mus *GAPDH*	F: AGGTCGGTGTGAACGGATTTG
	R: GGGGTCGTTGATGGCAACA

### Fluorescence *in situ* hybridization (FISH) assay

A dual color probe for PACSIN1 and the classical satellite III region of MHC-I (Zytovision, Bremerhaven, Germany) were used. The probe consists of a ZyGreen labeled probe, specific for PACSIN1, and a ZyOrange labeled CEN9 probe, specific for MHC-I. The nuclei were stained with DAPI. FISH was evaluated by two independent observers using a Nikon eclipse 80i microscope (Nikon) equipped with a Plan Apo VC 60× lens using oil immersion, and the hybridization was carried out in AGS and MKN-45 cells. Images were taken with a Leica DFC 450c camera system (Wetzlar, Germany) and Fix Foto software ( https://www.j-k-s.com).

### CCK-8 assay

Cell viability was determined using the CCK8 kit (GK10001; Glpbio, Montclair, USA) according to the manufacturer’s instructions. Briefly, the OT-I or MFC cells were plated into a 24-well plate (2×10 ^3^ cells/well). Then, the cells were incubated with CCK-8 (10 μL). Subsequently, the absorbance values were determined with a microplate reader (1681130; Bio-Rad, Hercules, USA) at a wavelength of 450 nm.

### Animal model

C57BL/6 male mice (6‒8 weeks, 18‒22 g) were obtained from the Animal Center of Nanjing Medical University (Nanjing, China). OT-I transgenic mice, *PACSIN1* ^‒/‒^ mice, and *Batf3* ^‒/‒^ mice were provided by The Jackson Laboratory (Bar Harbor, USA). All mice were housed at moderate temperature (22±2°C) with a humidity of 50%‒60% and a 12/12-h light/dark cycle, and had free access to food and water. All the animal experiment procedures were approved by the Animal Care Board of North Jiangsu People’s Hospital.

For orthotopic injection, mouse MFC cells (1×10 ^5^ cells) were suspended in 20 μL of Matrigel (356231; Corning, New York, USA):HBSS (1:1) solution and intraperitoneally injected into C57BL/6 mice. For liver metastasis assay, cells were suspended in 100 μL HBSS and then drawn into an insulin syringe (28-gauge needle, 329461; BD Biosciences, Franklin Lakes, USA), which was pre-loaded with 200 μL HBSS. The externalized spleen was divided by ligating clips (002200; Teleflex, Limerick, USA), and cells were injected into the hemispleen. After injection, splenic vein was ligated with ligating clips (001200; Teleflex) at the hilum of the spleen, and then the hemispleen was removed.

For the inhibition of CD8 ^+^ T cell infiltration, mice were intraperitoneally injected with 200 μg of anti-mouse CD8a antibody (BE0004-1; BioXcell, West Lebanon, USA) or anti-mouse IgG (BE0093; BioXcell). For ICB assay, mice were intraperitoneally injected with 200 μg of anti-PD-1 (BE0146; BioXcell) or anti-IgG (BE0093; BioXcell). The tumor volume was measured every three days. After 21 days, the mice were anesthetized with 35 mg/kg pentobarbital sodium (P3761; Sigma-Aldrich) and sacrificed using the cervical dislocation method. The tumor tissues of mice were isolated, photographed, and the tumor weight was recorded. The tissues were stored at ‒80°C for subsequent study.

### Hematoxylin-eosin (H&E) staining

Mouse gastric tissues were fixed in a 10% paraformaldehyde solution at 4°C, embedded in paraffin, and sectioned into 5-μm sections. The sections were deparaffinized with xylene and hydrated with gradient ethanol. H&E staining was then performed using standard techniques. The sections were incubated with a hematoxylin solution (ZLI-9610; OriGene Technologies, Inc., Beijing, China) for 2 min at room temperature (25°C) and washed with water for 6 min. Then, the sections were soaked in 70% ethanol solution containing 1% hydrochloric acid for 30 s at room temperature (25°C) and washed with water for 6 min again. Subsequently, they were stained with eosin (ZLI-9613; OriGene Technologies, Inc.) for 4 min at room temperature (25°C) and rinsed with water for 6 min. Finally, the stained sections were examined using an Olympus fluorescence inverted microscope (IX71; Olympus, Tokyo, Japan).

### OT-I cell isolation and culture

OT-I mice, C57BL/6-Tg (TcraTcrb) 1100Mjb/J, were purchased from The Jackson Laboratory. Mice were sacrificed by cervical dislocation and the spleen were removed. Spleen tissue (0.2 g) was homogenized using ultrasonic cell disruption system (GS-150Y; KUAN SON, Shanghai, China) in 1 mL RIPA Lysis Buffer (P0013B; Beyotime)and the homogenate was suspended in 2 mL red blood cell lysis buffer (11814389001; Sigma-Aldrich) for 1 min. The splenocytes were pelleted by centrifugation (200 *g*, 5 min)washed, and resuspended at 2×10 ^6^ cells/mL in RPMI culture medium containing 1 μg/mL OVA257-264 peptide (S7951; Sigma-Aldrich), 5 μg/mL recombinant mouse IL-2 (11271164001; Roche) and 40 μM 2-mercaptoethanol (63689; Sigma-Aldrich). The cells were incubated at 37°C for 5 days.

To set up the co-culture of OT-I and OVA ^+^ tumor cells, splenocytes were collected after 5 days of activation. OT-I cells were purified using EasySep mouse CD8 ^+^ T Cell Isolation kit (19663; Stemcell, Vancouver, Canada). MFC-OVA cells were cultured overnight. OT-I cells were then added into the culture at different time points. All cells were collected by trypsinization and analyzed by flow cytometry.

### Cytokine staining

Single-cell suspensions were prepared from fresh tumor tissues. T cells were enriched by density gradient centrifugation. For cytokine staining, T cells were incubated in culture medium containing PMA (5 ng/mL), ionomycin (500 ng/mL), brefeldin A (1:1000) and monensin (1:1000) at 37°C for 4 h. Then, anti-CD45 (30-F11), anti-CD90 (53-2.1), anti-CD4 (RM4-5), and anti-CD8 (53-6.7) antibodies obtained from Sigma-Aldrich were added and incubated for 20 min for surface staining. The cells were then washed and resuspended in 1 mL of freshly prepared Fix/Perm solution (BD Biosciences) and cultured at 4°C overnight. After being washed with Perm/Wash buffer (BD Biosciences), the cells were stained with anti-TNFα (MP6-XT22, SAB4502982; Sigma-Aldrich) and anti-IFNγ (XMG1.2, ZRB1620; Sigma-Aldrich) for 30 min, washed, and fixed in 4% formaldehyde (1.00496; Sigma-Aldrich). All samples were subject to cytometric analysis on an LSR II cytometer and analyzed with FACS DIVA software v. 8.0 (BD Biosciences).

### *In vitro* T-cell activation

Splenocytes were isolated from mice using a CD8 ^+^ T-cell Isolation kit (11814770001; Sigma-Aldrich). Then, the cells were plated into 24-well plates and incubated with anti-CD3/anti-CD28 antibodies (MA1-12098; Invitrogen, Carlsbad, USA) for 48 h in RPMI-1640 medium containing 10% FBS, 10 mM HEPES (SH30851.01; HyClone) 100 μM non-essential amino acids (60707ES60; YESEN), 50 U/mL penicillin-streptomycin solution (60162ES76; YESEN) and 50 μM β-mercaptoethanol (M3148; Sigma-Aldrich).

### Statistical analysis

Statistical analysis was performed using GraphPad 9.5.1 (San Diego, USA). Data are presented as the mean±SD. Student’s *t* test was carried out to analyze the differences between two groups, while one-way ANOVA was used for multigroup comparison. *P*<0.01 was considered to indicate statistical significance.

## Results

### Clinical characteristics of gastric patients

As shown in Table 2, the mean age of the gastric cancer patients was 53 years. Among the gastric cancer patients, 37 were diagnosed at an advanced stage, 42 had lymph node metastasis, and 41 had distant metastasis.

**Table:** **Table 2** Clinicopathological characteristics of the 62 patients

Variables	Number
Age (median, years)	53 (25–76)
Gender (male/female)	32/30
Histological diagnosis	
Tubular adenocarcinoma	20
Papillary adenocarcinoma	17
Mucinous adenocarcinoma	8
Poorly cohesive carcinoma	12
Signet-ring cell carcinoma	5
Disease stage	
I/II	25
III/IV	37
Lymph node metastasis	
N0	20
N1	19
N2	18
N3	5
Distance metastasis	
M0	21
M1	41
Recurrent	6

### High level of PACSIN1 is associated with poor prognosis in GC patients

**Figure 1:**
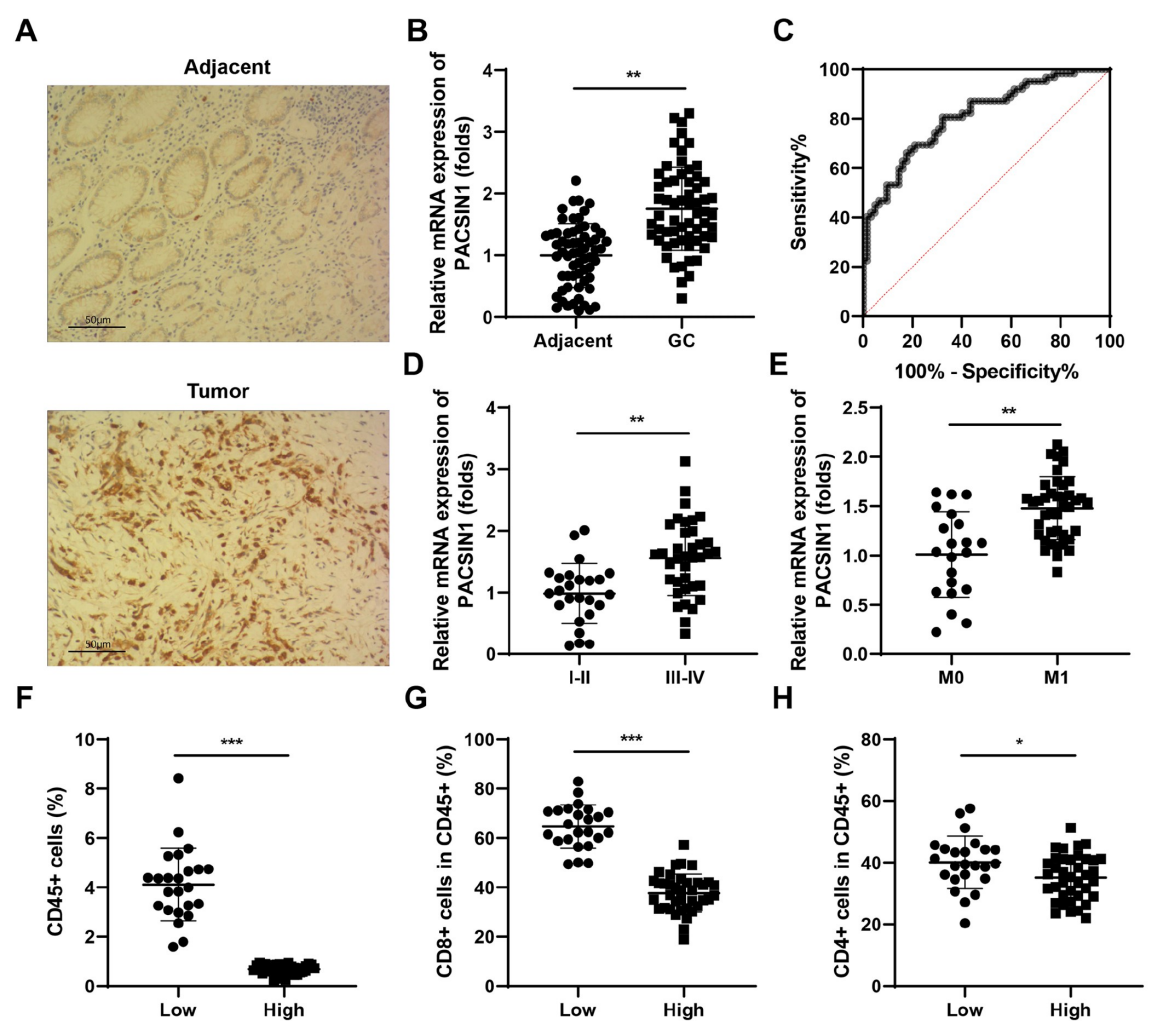
Figure 1 High level of PACSIN1 is associated with poor prognosis in GC patients (A) PACSIN1 expression in clinical samples was determined using immunohistochemistry. (B) PACSIN1 mRNA expression in clinical samples was determined using RT-qPCR. (C) ROC curve analysis of (B). (D–E) Subtype analysis of PACSIN1 mRNA expression in clinical samples. (F–H) The percentages of T cells were determined using flow cytometry in gastric cancer patients. * P<0.05, ** P<0.01, *** P<0.001.

The results from the IHC assay showed that the expression of PACSIN1 in tumor tissues was significantly greater than that in normal tissues ( Figure 1A). As shown in Figure 1B, *PACSIN1* mRNA expression was significantly increased in tumor tissues. ROC curve analysis (area: 0.83; 95% CI: 0.71‒0.92) showed that PACSIN1 can be a sensitive biomarker for GC patients ( Figure 1C). Additionally, a high level of PACSIN1 was associated with advanced disease stage ( Figure 1D) as well as distant metastasis ( Figure 1E). Moreover, in patients with high levels of PACSIN1, the infiltration of CD45 ^+^ T cells was markedly decreased ( Figure 1F). Additionally, the infiltration of CD8 ^+^ T cells was markedly decreased ( Figure 1G), whereas the infiltration of CD4 ^+^ T cells was only slightly decreased ( Figure 1H). *In vitro* assays also showed that *PACSIN1* knockout strongly affected the proliferation of CD8 ^+^ T cells and enhanced the cytotoxicity of CD8 ^+^ T cells ( Supplementary Figure S1). These findings suggested that *PACSIN1* may function as an oncogene in GC by inhibiting the function of CD8 ^+^ T cells.

### PACSIN1 promotes MHC-I trafficking to the lysosome

**Figure 2:**
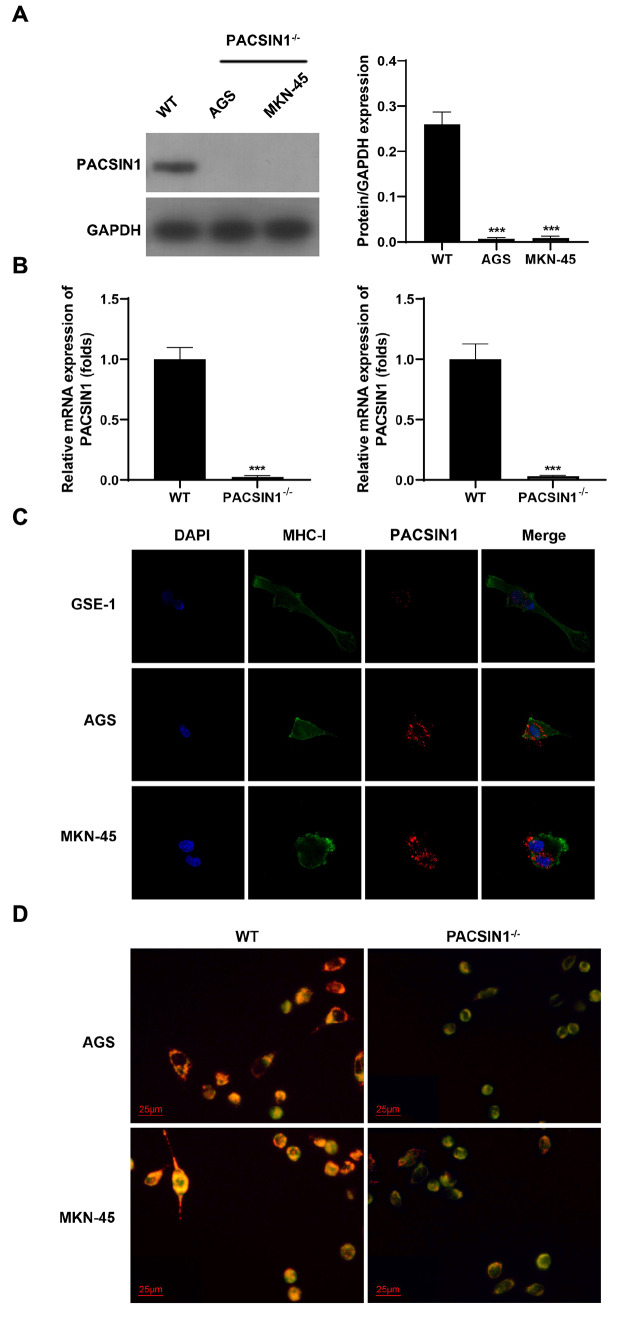
Figure 2 PACSIN1 promotes MHC-I trafficking to the lysosome (A) PACSIN1 protein expression in AGS and MKN-45 cells was determined by western blot analysis. (B) PACSIN1 mRNA expression in AGS and MKN-45 cells was determined by RT-qPCR after knockout of PACSIN1. (C) The co-localization of PACSIN1 and MHC-I was determined by FISH assay in AGS, MKN-45 and GSE-1 cells. (D) The co-localization of PACSIN1 and MHC-I was determined using FISH assay in PACSIN1-knockout AGS and MKN-45 cells. *** P<0.001.

Antigen-specific CD8 ^+^ T-cell responses depend on MHC-I, which is the “guide” for cytotoxic CD8 ^+^ T cells to find cancer cells [ 24– 26] . To further determine the roles of PACSIN1 in the antitumor T-cell immunity of GC, we generated *PACSIN1* ^‒/‒^ cells using AGS and MKN-45 cells and determined the expression of MHC-I in gastric cancer. PACSIN1 protein and mRNA expressions were markedly decreased in the *PACSIN1*-knockout group ( Figure 2A,B). PACSIN1 and MHC-I were more frequently co-localized in AGS and MKN-45 cells than in GES-1 cells ( Figure 2C). Moreover, PACSIN1 and MHC-I were localized in the lysosomes of AGS and MKN-45 cells ( Figure 2D).

### PACSIN1 inhibits tumor cell antigen presentation in GC

**Figure 3:**
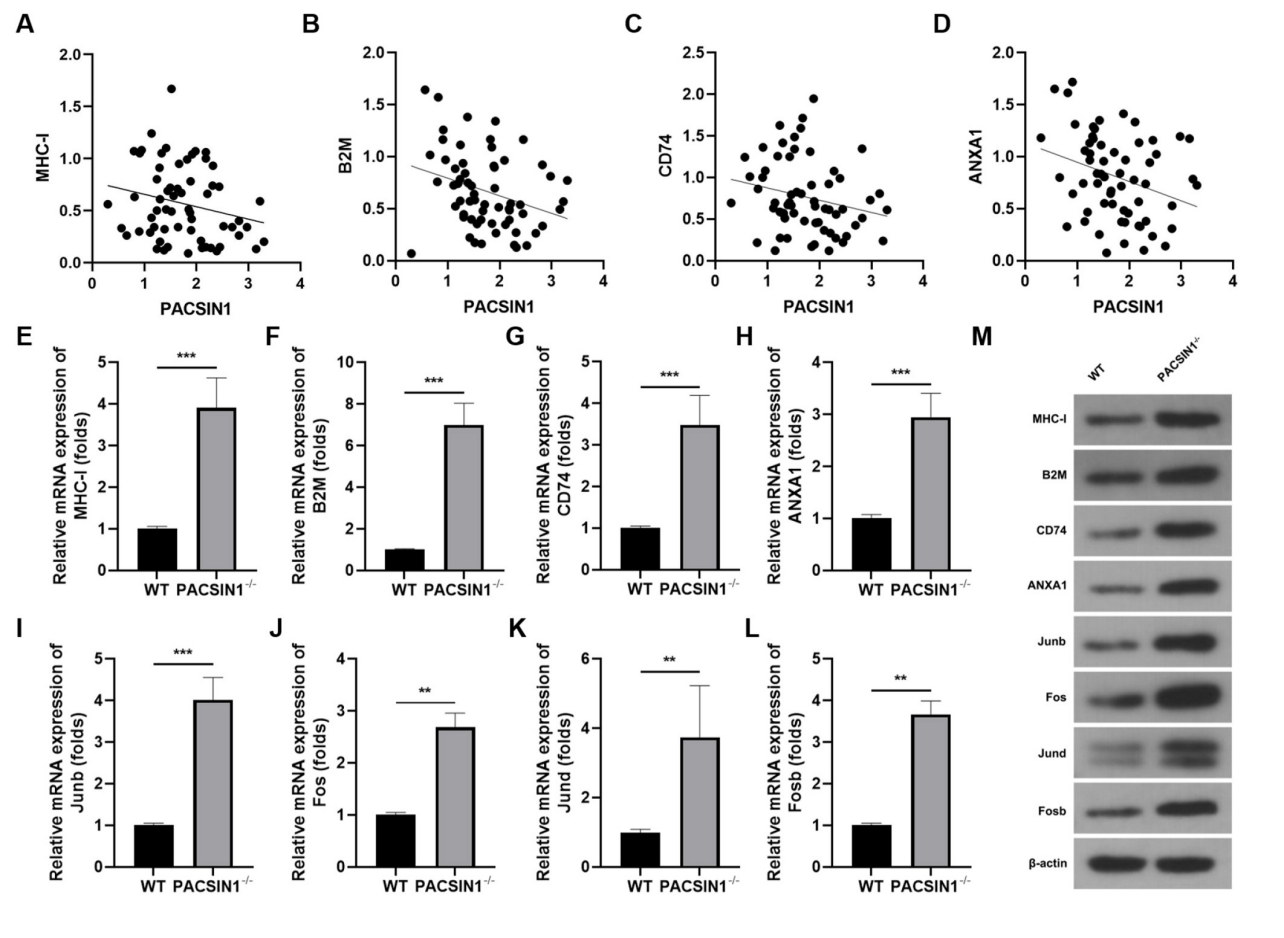
Figure 3 PACSIN1 inhibits tumor cell antigen presentation in GC (A–D) The mRNA expressions of MHC-I, B2M, CD74, and ANXA1 in clinical samples were determined by RT-qPCR. (E‒L) The mRNA expressions of MHC-I, B2M, CD74, ANXA1, Junb, Fos, Jund, and Fosb in GC tissues formed by normal MFC cells or PACSIN1 ‒/‒ MFC cells was determined by RT-qPCR. (M) The protein expressions of MHC-I, B2M, CD74, ANXA1, Junb, Fos, Jund, and Fosb in GC tissues formed by normal MFC cells or PACSIN1 ‒/‒ MFC cells were determined by western blot analysis. ** P<0.01, *** P<0.001.

We found that the mRNA expressions of *MHC-I*, *B2M*, *CD74*, and *ANXA1* were significantly decreased in GC patients with high PACSIN1 expression ( Figure 3A‒D). In *PACSIN1* ^‒/‒^ MFC cells, the mRNA and protein expressions of MHC-I, B2M, CD74, ANXA1, Junb, Fos, Jund, and Fosb were significantly increased ( Figure 3E‒M).

### *PACSIN1* knockout enhances the killing ability of CD8 ^+^ T cells

**Figure 4:**
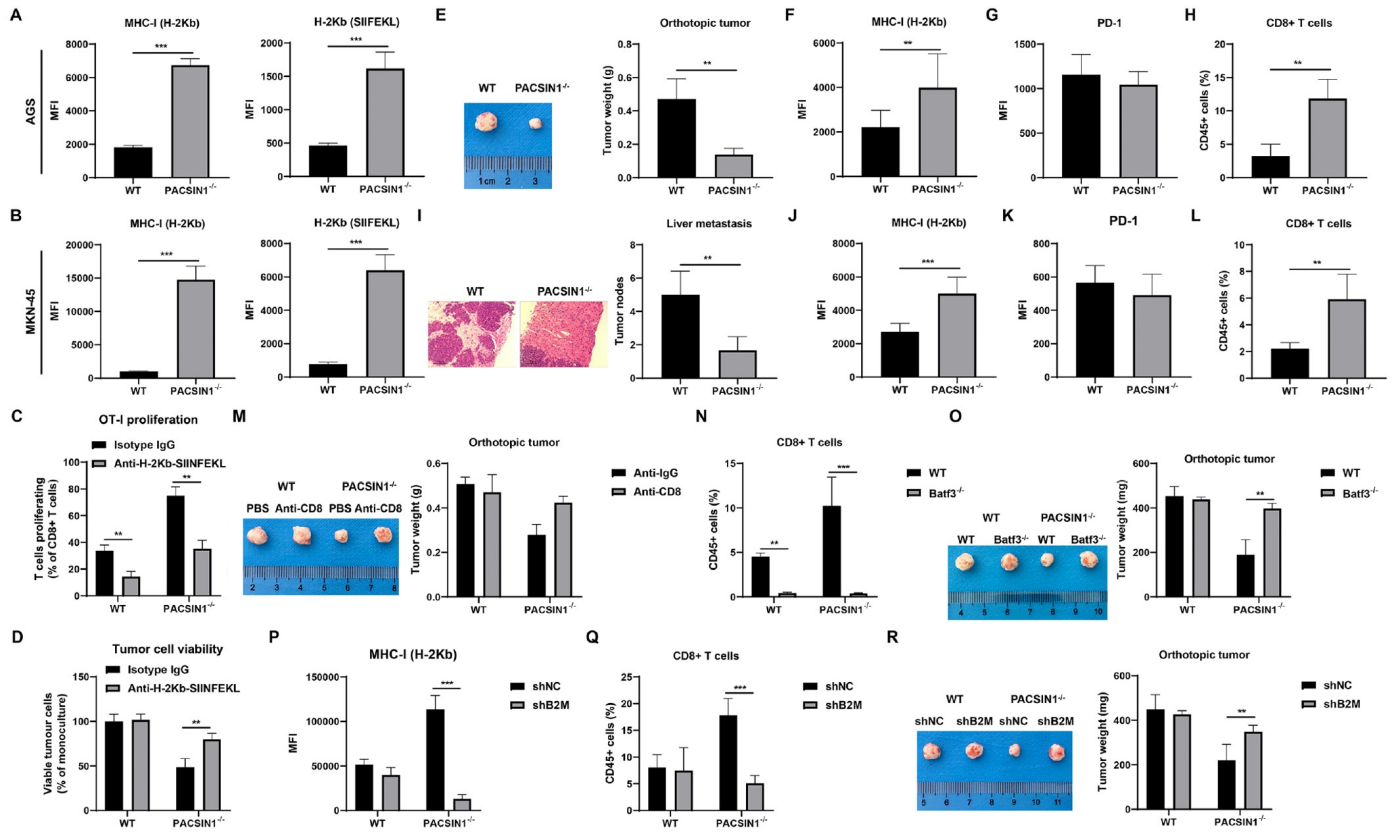
Figure 4 *PACSIN1* knockout enhances the killing ability of CD8 ^+^ T cells (A,B) MHC-I and H-2Kb-SIINFEKLs levels in AGS and MKN-45 cells were determined by flow cytometry. (C) OT-I proliferation was determined by flow cytometry assay. (D) AGS cell viability was determined using a CCK-8 assay. (E) The tumor size. (F,G) The levels of MHC-I (F) and PD1 (G) in GC tissues formed by normal AGS cells or PACSIN1 ‒/‒ AGS cells were determined by flow cytometry. (H) The number of tumor-infiltrating CD8 + T cells was determined by flow cytometry. (I) PACSIN1 knockout inhibited liver metastasis. (J,K) The levels of MHC-I (J) and PD1 (K) in liver nodes were determined by flow cytometry. (L) The number of tumor-infiltrating CD8 + T cells was determined by flow cytometry. (M) The tumor size. (N) The number of tumor-infiltrating CD8 + T cells was determined by flow cytometry. (O) The tumor size. (P) The level of MHC-I was determined by flow cytometry. (Q) The number of tumor-infiltrating CD8 + T cells was determined by flow cytometry. (R) The tumor size. ** P<0.01, *** P<0.001.

*PACSIN1* knockout increased the surface expressions of both MHC-I and the OVA-derived peptide SIINFEKL bound to H-2Kb ( Figure 4A,B), confirming enhanced peptide presentation. After co-culture with OT-I GC cells, *PACSIN1* knockout significantly increased the proliferation of CD8 ^+^ T cells ( Figure 4C). Moreover, *PACSIN1* knockout significantly inhibited the viability of MFC cells after co-culture with OT-I cells ( Figure 4D). However, the killing ability of CD8 ^+^ T cells was MHC-I-dependent because treatment with specific H-2Kb antibodies markedly suppressed the proliferation of OT-I cells and increased the viability of MFC cells compared to those in the *PACSIN1*-knockout group ( Figure 4C,D).

*PACSIN1* knockout markedly decreased tumor size and weight ( Figure 4E) and increased the expression of MHC-I ( Figure 4F) but did not obviously change PD-1 expression ( Figure 4G). *PACSIN1* knockout also increased the infiltration level of CD8 ^+^ T cells ( Figure 4H). This finding was in line with that in mice with liver metastasis. As shown in Figure 4I–L, the number of liver nodes was significantly decreased in *PACSIN1*-knockout mice, as indicated by the increase in MHC-I expression and CD8 ^+^ T-cell infiltration but no significant alteration in PD-1 expression.

Notably, antibody-mediated CD8 ^+^ T-cell depletion restored tumor size and weight in *PACSIN1*-knockout mice ( Figure 4M). Moreover, *PACSIN1* knockout-mediated CD8 ^+^ T-cell infiltration was alleviated in *Batf3* ^‒/‒^ mice ( Figure 4N). Accordingly, *PACSIN1* knockout-mediated inhibition of GC cell growth was restored in *Batf3* ^‒/‒^ mice ( Figure 4O), suggesting that blocking the interaction between MHC-I and PACSIN1 may enhance the tumor cell recognition of CD8 ^+^ T cells.

Subsequently, to verify the potential role of MHC-I in GC, MHC-I expression was inhibited by *B2M* knockdown. *B2M* knockdown significantly decreased MHC-I level ( Figure 4P) and decreased CD8 ^+^ T-cell infiltration ( Figure 4Q) but promoted tumor size and weight ( Figure 4R). The above findings suggested that the MHC-I-mediated increase in antigen presentation in GC cells after *PACSIN1* knockout is a prerequisite for promoting CD8 ^+^ T-cell-mediated antitumor immunity.

### *PACSIN1* knockout sensitizes GC cells to ICB

**Figure 5:**
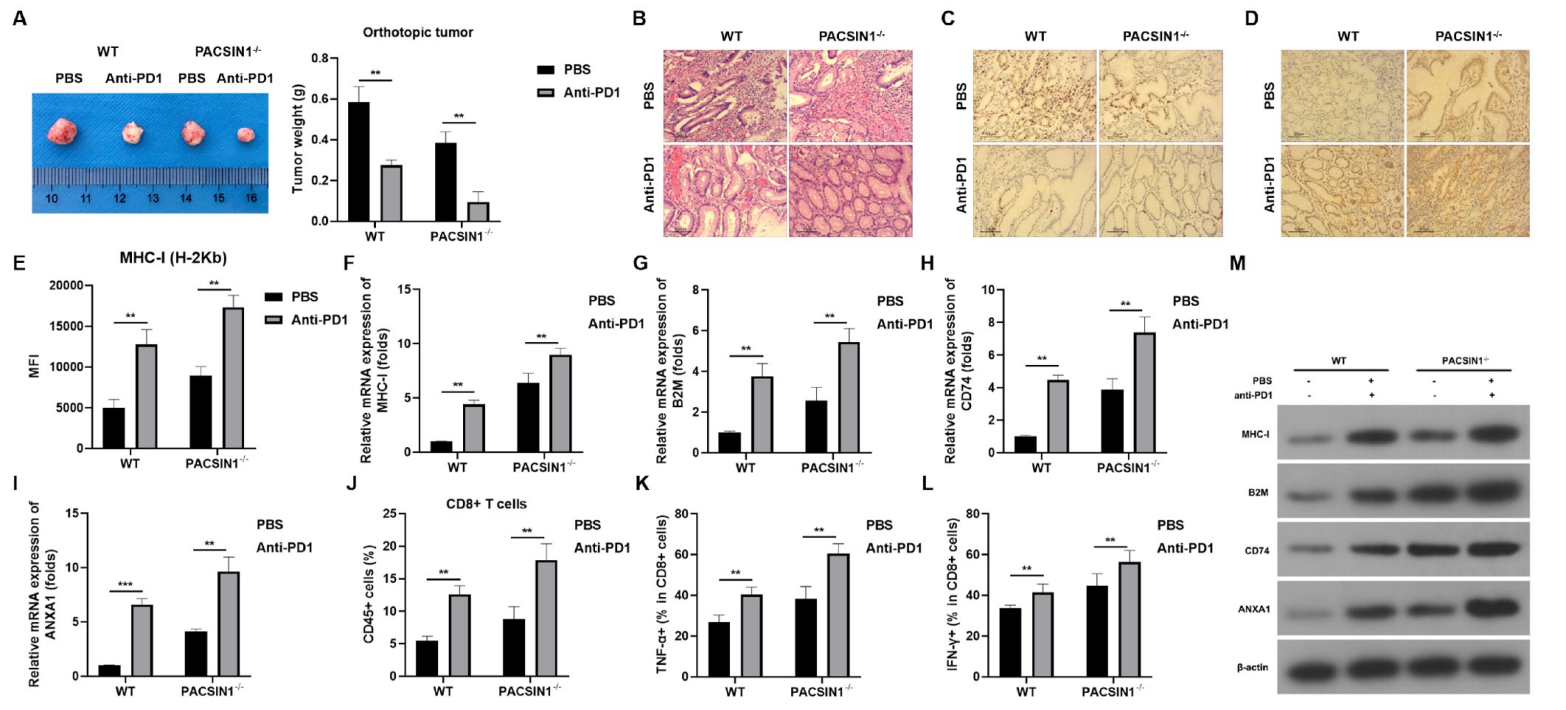
Figure 5 *PACSIN1* knockout sensitizes GC cells to ICB (A) The tumor size. (B) Histological analysis was performed using H&E staining. (C,D) The expressions of PD-1 (C) and MHC-I (D) in GC tissues were determined using IHC. (E) H-2Kb levels in GC tissues were determined by flow cytometry. (F–I) The mRNA expressions of MHC-I (F), B2M (G), CD74 (H), and ANXA1 (I) in GC tissues were determined by RT-qPCR. (J) The infiltration level of CD8 + T cells was determined using flow cytometry. (K,L) The release of TNFα (K) and IFNγ (L) in GC tissues was determined by flow cytometry. (M) The protein expressions of MHC-I, B2M, CD74, and ANXA1 in GC tissues was determined by western blot analysis. ** P<0.01.

The acquired chemoresistance of GC is the main cause of GC-related death. Therefore, we determined whether *PACSIN1* knockout could promote the chemosensitivity of GC to ICB via syngeneic injection of *PACSIN1* ^‒/‒^ and anti-PD1 antibodies. As shown in Figure 5A, *PACSIN1* knockout or anti-PD1 therapy significantly inhibited GC tumor size and weight. Moreover, syngeneic injection had more pronounced effects. Histological analysis revealed that *PACSIN1* ^‒/‒^ or anti-PD1 injection alleviated the invasion of the serosa in the sham group ( Figure 5B). Furthermore, *PACSIN1* knockout enhanced the antitumor effects of anti-PD1 therapy, as indicated by the nearly normal level of gastric epithelial rearrangement and maintenance of integrity. *PACSIN1* knockout also significantly inhibited the expression of PD-1 ( Figure 5C) but increased the expression of MHC-I ( Figure 5D), suggesting that *PACSIN1* knockout may promote the immunotherapy of anti-PD1, inhibiting tumor growth and possibly promoting GC metastasis.

We also analyzed the tumor microenvironment (TME) after *PACSIN1* ^‒/‒^ and/or anti-PD-1 antibody injection. *PACSIN1* knockout significantly enhanced the effects of anti-PD1 therapy. PACSIN1 combined with anti-PD1 also significantly increased the level of H-2Kb ( Figure 5E) as well as the mRNA and protein expressions of MHC-I, B2M, CD74, and ANXA1 in GC tissues ( Figure 5F‒I,M). Compared with single therapies, PACSIN1 combined with anti-PD1 significantly increased the infiltration level of CD8 ^+^ T cells ( Figure 5J). Moreover, the release of TNFα and IFNγ in CD8 ^+^ T cells was significantly increased by *PACSIN1* knockout combined with anti-PD1 therapy ( Figure 5K‒L). These findings suggested that *PACSIN1* knockout may promote the sensitivity of GC to anti-PD1 by activating CD8 ^+^ T cells.

## Discussion

The present study revealed the immune evasion of GC cells mediated by lysosomal fusion of MHC-I in. Our finding is consistent with that of Ren *et al*. [27], who demonstrated that MHC-I downregulation-induced inactivation of CD8 ^+^ T cells contributes to immune silencing in GC. PACSIN1 is a newly identified autophagy regulator [28] that is indispensable for lysosomal fusion. The PACSIN1-mediated selective autophagy of MHC-I induced its degradation in the membranes of GC cells, which was correlated with decreased infiltration of CD8 ^+^ T cells. Considering the key role of autophagy and the lysosomal system in the TME of GC [ 29, 30] , our study may provide novel insight into immunologically-cold tumors.

PACSIN1 is frequently found to be abnormally expressed in multiple types of cancers. For instance, overexpression of PACSIN1 in luminal breast cancer predicts poor prognosis and promotes the aggressiveness of breast cancer cells [22], suggesting that *PACSIN1* may function as an oncogene in luminal breast cancer. However, reports on the roles of PACSIN1 in cancers are contradictory. A low level of PACSIN1 in brain glioma patients predicts advanced disease stage and unsatisfactory long-term overall survival [21], suggesting that *PACSIN1* may act as an antitumor gene in brain glioma. Therefore, the roles of PACSIN1 may vary with the type of cancer. Therefore, identifying the potential of PACSIN1 is of vital importance. In this study, PACSIN1 was found to be overexpressed in GC patients, especially in immunologically-cold tumors. Moreover, high expression of PACSIN1 was associated with advanced stages, lymph node metastasis and poor prognosis. These findings suggested that *PACSIN1* may function as an oncogene in GC, which is in line with the findings of Liu *et al*. [23]. PACSIN1, an autophagy regulator, promotes lysosomal fusion and autophagy. PACSIN1-mediated lysosomal fusion and selective autophagy of MHC-I contribute to the immune evasion of GC cells. Apart from PACSIN1-mediated effects on type I IFN signaling cascades in plasmacytoid dendritic cells [17], PACSIN1 limited the ability of CD8 ^+^ T cells to recognize GCs and suppressed CD8 ^+^ T-cell infiltration, suggesting that PACSIN1 may exert its oncogenic effects by suppressing antitumor T-cell immunity.

In this study, we investigated the potential of CD8 ^+^ T cells in GC because CD8 ^+^ T cells can recognize tumor cells by directly interacting with MHC-I. Antigen presented by the cancer cell membrane protein MHC-I stimulates the activation of TCR/CD3/B7 signaling, which co-activates CD8 ^+^ T cells to recognize and kill tumor cells [31]. These findings suggested that MHC-I not only determines the specificity of CD8 ^+^ T cells but also is essential for their activation and proliferation [ 24, 32, 33] . However, the degradation of MHC-I in tumor cell membranes prevents the presentation of antigens, resulting in the evasion of tumor cells [34]. In this study, lysosomal fusion and selective autophagy of MHC-I were found to induce its degradation in the membranes of GC cells, which was accompanied by a decrease in the infiltration level of CD8 ^+^ T cells. Therefore, targeting autophagy inhibition may be a promising strategy for stimulating antitumor T-cell immunity in GC.

Recent studies have demonstrated the potential of autophagy in cancer. On one hand, autophagy provides nutrients for tumor cell growth and proliferation [35]; on the other hand, lysosome formation inhibits exosome secretion and tumor cell metastasis [36]. Given the “double-dealer” nature of autophagy in tumors, a “one size fits all” strategy for inhibiting or enhancing autophagy in cancer treatment is impractical. However, in immunologically-cold tumors, inhibiting autophagy is generally demonstrated to be a promising strategy [ 37, 38] . This may be because the inhibition of autophagy promotes antigen presentation and recognition, as well as immune cell infiltration [ 39, 40] . In this study, *PACSIN1* deficiency inhibited lysosomal fusion and selective autophagy of MHC-I and promoted antigen presentation and infiltration of CD8 ^+^ T cells, which suppressed tumor growth and liver metastasis and enhanced the chemosensitivity of GC to anti-PD1 therapy *in vivo*. Currently, genetic mutations and/or loss of expressions in genes that modulate the stages of MHC-I antigen presentation are associated with acquired chemoresistance to ICB [24]. Therefore, targeting PACSIN1 may be an alternative strategy for promoting the chemosensitivity of GC cells to ICBs.

In conclusion, *PACSIN1* may function as an oncogene in GC. The PACSIN1-mediated lysosome fusion and selective autophagy mediated by MHC-I inhibited antigen presentation and infiltration of CD8 ^+^ T cells, contributing to immunologically-cold tumors in GC. Therefore, targeting PACSIN1 may be a promising strategy for activating antitumor immunity in GC.

## Supporting information

Supplementary_Figure_S1

## Supplementary Data

Supplementary data is available at *Acta Biochimica et Biophysica Sinica* online.
