# Supplementary material for: PACSIN1 promotes immunosuppression in gastric cancer by degrading MHC-I: The roles of PACSIN1 in gastric cancer
Source: Acta Biochim Biophys Sin (Shanghai). 2024 May 31;56(10):1473–82. doi: 10.3724/abbs.2024059 (PMC11532212; doi:10.3724/abbs.2024059)
Supplement: Supplementary_Figure_S1 [file Supplementary_Figure_S1.pdf]

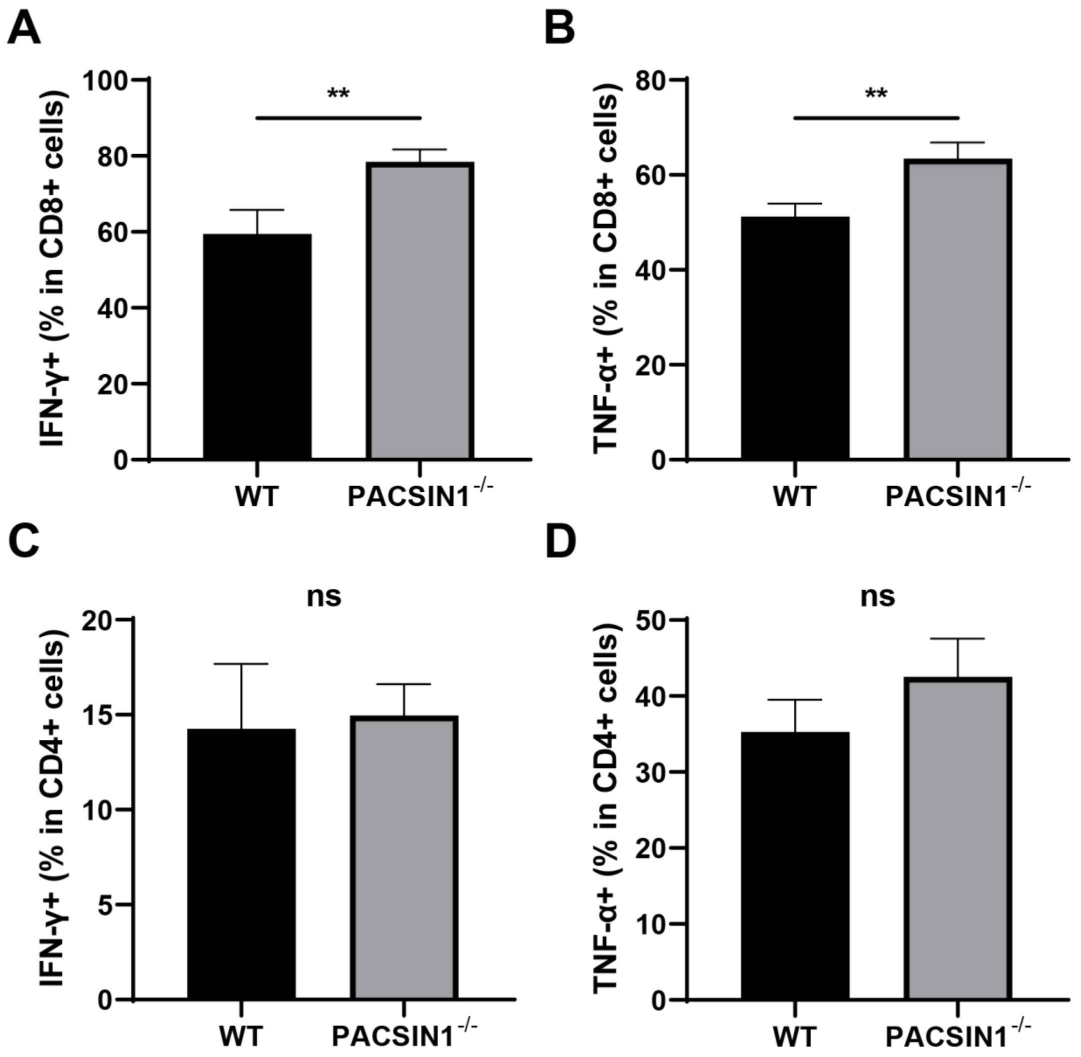

Supplementary Figure S1. The functions of CD8$^+$ T cells and CD4$^+$ T cells

(A–D) The functions of CD8+ T cells and CD4+ T cells were determined using flow cytometry. ***P*<0.01.
